# Development and Validation of the Companion's Satisfaction Questionnaire of Patient's Hospitalized in Intensive Care Units

**DOI:** 10.17533/udea.iee.v42n1e09

**Published:** 2024-04-28

**Authors:** Ali Dehghani

**Affiliations:** 1 Associate professor, Department of Community Health Nursing, School of Nursing, Jahrom University of Medical Sciences, Jahrom, Iran. Email: ali.dehghani2000@gmail.com. Corresponding author Jahrom University of Medical Sciences Department of Community Health Nursing School of Nursing Jahrom University of Medical Sciences Jahrom Iran ali.dehghani2000@gmail.com

**Keywords:** validation study, personal satisfaction, surveys and questionnaires, nursing care, intensive care units., estudio de validación, satisfacción personal, encuestas y cuestionarios, atención de enfermería, unidades de cuidados intensivos., estudo de validação, satisfação pessoal, inquéritos e questionários, cuidados de enfermagem unidades de terapia intensiva.

## Abstract

**Objective.:**

The current study aimed to develop and validate of companions’ satisfaction questionnaire of patients hospitalized in ICUs.

**Methods.:**

This is a methodological study that was performed in three phases: In the first phase, the concept of companion's satisfaction of patients hospitalized in ICUs was defined through qualitative content analysis method. In the second phase, early items of questionnaire were generated based on findings of the first phase. In the third and final phase, validation of the questionnaire was evaluated using face, content and construct validity as well as reliability.

**Results.:**

In exploratory factor analysis, three subscales including: satisfaction with nursing staff communication (5 items), satisfaction with nursing care (12 items), and satisfaction with decision making (5 items) were extracted by Eigen value above one and factor load above 0.5. Internal consistency and stability of the developed questionnaire confirmed with 0.94 and 0.95 respectively that indicated acceptable reliability.

**Conclusion.:**

The 22-item developed questionnaire is valid and reliable for measurement of levels of companion's satisfaction of Iranian patients hospitalized in ICUs.

## Resumen

**Objetivo.:**

Desarrollar y validar un cuestionario de satisfacción de acompañantes de pacientes hospitalizados en UCIs.

**Métodos.:**

Estudio de validación que se realizó en tres fases: en la primera se definió el concepto de satisfacción de los acompañantes de pacientes hospitalizados en UCI mediante el método de análisis de contenido cualitativo; en la segunda fase se generaron los primeros ítems del cuestionario a partir de los resultados de la primera fase; y en la tercera fase se evaluó la validación del cuestionario mediante la validez facial, de contenido y de constructo, así como la fiabilidad.

**Resultados.:**

En el análisis factorial exploratorio, se extrajeron tres subescalas que incluían: satisfacción con la comunicación del personal de enfermería (5 ítems), satisfacción con los cuidados de enfermería (12 ítems) y satisfacción con la toma de decisiones (5 ítems) con un valor Eigen superior a uno y una carga factorial superior a 0.5. La consistencia interna y la estabilidad del cuestionario desarrollado se confirmaron con 0.94 y 0.95 respectivamente, lo que indicaba una fiabilidad aceptable.

**Conclusión.:**

El cuestionario desarrollado de 22 ítems es válido y fiable para medir los niveles de satisfacción de los acompañantes de pacientes iraníes hospitalizados en UCI.

## Resumo

**Objetivo.:**

Desenvolver e validar um questionário sobre a satisfação dos acompanhantes de pacientes hospitalizados em UTIs.

**Métodos.:**

Estudo de validação realizado em três fases: na primeira fase, o conceito de satisfação de acompanhantes de pacientes internados em UTIs foi definido por meio do método de análise qualitativa de conteúdo; na segunda fase, os primeiros itens do questionário foram gerados a partir dos resultados da primeira fase; e na terceira fase, a validação do questionário foi avaliada por meio da validade de face, de conteúdo e de construto, bem como da confiabilidade.

**Resultados.:**

Na análise fatorial exploratória, três subescalas foram extraídas, incluindo: satisfação com a comunicação da equipe de enfermagem (5 itens), satisfação com a assistência de enfermagem (12 itens) e satisfação com a tomada de decisões (5 itens) com um valor Eigen maior que um e uma carga fatorial maior que 0.5. A consistência interna e a estabilidade do questionário desenvolvido foram confirmadas com 0.94 e 0.95, respectivamente, indicando confiabilidade aceitável.

**Conclusão.:**

O questionário de 22 itens desenvolvido é válido e confiável para medir os níveis de satisfação dos acompanhantes de pacientes iranianos hospitalizados em UTI.

## Introduction

In recent years, quality of care has become an important matter in healthcare systems worldwide. Particularly, the quality of care as perceived by patients and their relatives is a current focus of interest.^1^ Satisfaction with nursing cares is one of the indicators of quality of care in health centers in all countries. Therefore, people satisfaction has been concerned for health care and hospitals managers.^2^^,^^3^ In deed the key to the success of any health center or hospital is to obtain satisfaction with nursing care and to improve patient's satisfaction.^4^ Over the last three decades, investigations on health service delivery and patient satisfaction have increasingly played important roles as quality indicators to improve and evaluate the consequences of care provided by health care centers.^5^ Hence, measuring satisfaction with nursing care is essential to assess the outcome of ongoing efforts to improve quality of care and ensure hospital progress.^6^ In Iran, since 2011, the Ministry of Health and Medical Education, in line with its main mission, all hospitals have obliged to periodically assess patient satisfaction and interventions needed to increase patient satisfaction.^7^

Most often we have problem with measuring patients ‘satisfaction in ICUs.^8^ Many patients in ICUs are critically unwell, sedated, paralyzed, and unable to communicate. As such, the viewpoints of families and companion's become highly relevant.^5^^,^^9^ Patients in ICUs even may not remember critical care experience completely, which is very important in investigating patients ‘satisfaction.^10^ Because most ICU patients cannot make decisions themselves, family members and companions are actively involved in the care process as surrogate decision-makers and are, hence, judges about quality of care. However, family and companions’ satisfaction with care is complex and is not clearly defined.^11^^,^^12^ Therefore, patient's companion's satisfaction can be measured as a substitute for patients ‘satisfaction in these units. Patients' companion's is a part of taking care of the patients, as well as providing support for the patients' family and companions can affect patients' improvement.^13^ Consequently, assessing family and companions needs and satisfaction with care and information/decision making must be an integral part of quality assessment in the ICU.^14^ Satisfaction is a balance of expectations and actual care delivered and heavily dependent on societal perception of adequate care.^15^

Measuring companion's satisfaction of the patients hospitalized in ICUs requires standard and context-based questionnaires. First reports of families’ viewpoints date from the 1970s.^16^ However, only recently have tools been validated- e.g., the CCFSS^17^ and the FS-ICU^18^^,^^19^ that systematically measure family satisfaction. This tools are being extensively used in other countries. But measuring the satisfaction of patients companions admitted to ICUs requires specific and context - based tools tailored to socio-cultural conditions that can provide accurate data about the quality of nursing care in that particular country.

The only study in this regard in Iran is a research conducted by Dolatyare *et al.*^20^ On the translation and localization of the 34-item satisfaction questionnaire of family patients in ICUs Canadian version (FS-ICU 34) which after translating and performing face, content, and construct validity to 30 items has been reduced in three dimensions: satisfaction with medical staff performance (12 items), comfort (12 items) and decision making (6 items). In designing this questionnaire, the opinions and perspectives of patients' companions and families in Iran for generation of items and design of questionnaire were not taken into consideration and only the questionnaire designed in another country has been translated and localized. Because patient's companion's satisfaction can be influenced by several factors, and the acquired data must be accurate, good validation is obligatory for the adequate use of the questionnaires. Psychometric properties like validity and reliability, are essential components of questionnaires due to these describe the quality of the measurement. Questionnaires lacking acceptable validation may not measure the construct they intend to assess, or the values that arise from the questionnaire may not indicate the “true” value.^1^^,^^21^ This may not only disrupt research but as well as misguide the health team working with the questionnaire. Therefore, the quality of a questionnaire is evaluated by its psychometric properties and rate of symmetry with social and cultural structure of target community.^1^^,^^22^Therefore, the aim of the present study was to develop a valid and reliable questionnaire that assesses level of satisfaction of patient's companions in ICUs (CS-ICU).

## Methods

Study design and participants. This study was a methodological study that was performed in three phases. Data in this study were collected from April 2022 to July 2023 at the educational hospitals in Jahrom, Iran. Patient's companions included family members and relatives. 

**Figure 1:**
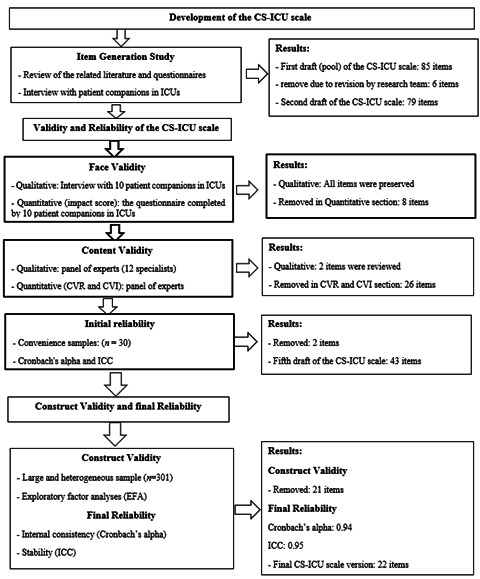
Flow diagram of the development and validation of the companion's satisfaction questionnaire of patients hospitalized in ICUs (CS-ICU scale)

Phases of the study. This research was performed in three phases as follows (Figure 1): 

*The first phase.* In this phase, the concept of patient's companion's satisfaction in ICUs was conceptualized and defined by the qualitative content analysis method. In this method, the codes and their categories were directly extracted from the interviews. In qualitative content analysis, the researcher interpreted the results using presenting data in Microsoft words and categories and dimensions which involved understanding, interpreting and conceptualizing of the underlying meanings of the qualitative data.^23^ In this part, 25 patient's companions in ICUs participated in the research. The collection data were conducted through semi - structured and in- depth interviews. Inclusion criteria of patient's companions were (1) willingness to participate in the study, (2) ability to express experiences, (3) passing at least 48 hours of admission in ICU, (4) the presence of the patient's companions including close relatives and those who make decision for the patient including spouse, father, mother, sister, brother, friend, and his/ her children, (5) visiting the patient at least three times in ICU. Exclusion criteria of participants were (1) patient's companions younger than 18 years, (2) patient's companions with cognitive impairment and mental disabilities, and (3) lack of patient's companion's willingness to continue the study. Each interview lasted on average 40 - 60 min. A total interview was conducted in hospital in Jahrom based on their prior agreement and at the time they were comfortable. Interview with patient's companions continued to data saturation. Interviews were tape-recorded, transcribed verbatim in Microsoft words software, Ver2013 for manage the coding process. Then, analysis data were conducted using qualitative content analysis and Graneheim and Lundman approach.^24^ In this stage, the primary codes were extracted and the subcategories and categories were formed. In the end of this part, the dimensions of patient's companion's satisfaction in ICUs were extracted and provided a final definition of the concept of patient's companion's satisfaction in ICUs.

***The second phase*.** In this part, the items pool of was formed for design a patient's companion's satisfaction questionnaire in ICUs according to the following stages: (i) Dimensions extracted from the first phase of the study for patient's companion's satisfaction in ICUs; (ii) Reviewing relevant texts and articles regarding patient's companion's satisfaction in ICUs, and (iii) Reviewing relevant questionnaires in the field of patient's companion's satisfaction in ICUs.

*The third phase.* In this phase, psychometric properties of developed questionnaire were evaluated. These properties included face, content, and construct validity, and reliability questionnaire. These properties were distributed the following:

a) Face validity: The face validity was conducted in the two qualitative and quantitative sections. The qualitative section was performed through interviews with 10 patient's companions in ICUs. The patient's companions about difficulty, suitability and ambiguous of the questionnaire items were asked and their recommendations on the items were applied. In the quantitative section, the impact score was calculated for the importance every item and remove inappropriate items. Thus, for every item within the questionnaire, a Likert scale with 5 - Likert points scale and scores of 1-5 was considered and rated. The range of options include: very important (score 5), important (score 4), standard importance (score 3), slightly important (score 2), and not important (score 1). Then, the developed questionnaire was completed by 10 patient's companions in ICUs. Impact score was achieved above 1.5 for all the items in this part.^25^ The method used to calculate the Impact Score was: *Impact Score=Frequency (%) × Importance* (Importance= patient's companions who have checked options 4 and 5). ^26^

b) Content validity: The content validity was conducted in the two qualitative and quantitative sections. In qualitative part, 12 experts were asked to assess the questionnaire about grammar, using appropriate words, placement of items in the appropriate place and right scoring.^27^ In quantitative part, content validity ratio (CVR) and content validity index (CVI) were determined for every item of questionnaire. For evaluating the necessity of every items of the questionnaire, the CVR according to Lawshe^28^ scale and modified table by Ayre and John Scally^29^ was used. Based on the Lawshe scale the CVR was calculated on a three-point scale. Every item was scored according to three options on the graph (1=not necessary, 2=useful, but not essential, and 3=essential) by 10 experts. If the CVR score is higher than 0.80, the CVR of the scale has been approved.^26^^,^^28^ The method used to calculate the CVR was:




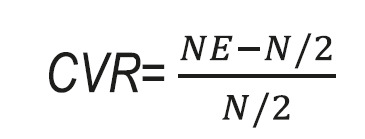




Where N= the total number of specialists and NE=the number of specialists who have checked option 3. In the CVI, the relevance of each item was analyzed by 10 experts on a four-point Likert scale (not relevant: 1; a little relevant: 2; somewhat relevant: 3; and extremely relevant: 4).^30^ The acceptable and adequate amount for the CVI was equal to 0.79 and if the CVI for every item was calculated to be less than 0.79 it would be considered unacceptable and that item would be eliminated from the questionnaire.^31^ If the CVI scores for every item was between 0.70-0.79 that item is questionable and challengeable and so requires further revision and modifications.^26^^,^^31^ The method used to calculate the CVI was:




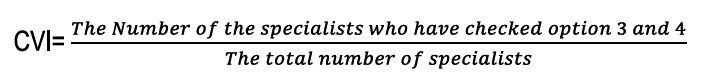




c) Initial reliability: In this section, correlation coefficient between items and as well as between items and whole questionnaire were determined using the Cronbach's alpha and inter-item correlation coefficient (ICC) by 30 patient companions in ICUs.

d) Construct validity: in this part, exploratory factor analysis (EFA) was used to determine the construct validity of the CS-ICU scale. The EFA was used to determine the interrelationship between items and to summarize related items in a dimension.^32^ In the EFA from the principal component analysis (PCA) for factors extraction, Kaiser- Meyer- Olkin index (KMO) for determine sampling adequacy, Bartlett's Test for evaluation the correlation between the items of the questionnaire in order to integrate them and varimax rotation for simplify and interpret the factor structure using taking the Eigen value above one was used. In addition to, the scree plots as well as for determination the number of factors was used. The number of people required for carrying out factor analysis per every item between 3 - 10 samples.^33^ Thus, in the present study, the CS-ICU scale was completed by 301 patient companions in ICUs using convenience sampling. The factor loading for every item in order to item maintenance above 0.5 was considered.

e) Final reliability: Reliability of the CS-ICU scale was calculated through two internal consistency and stability methods. For calculate the internal consistency, the CS-ICU scale was completed by 30 patient companions in ICUs and then Cronbach's alpha coefficient was determined. Alpha coefficient at least 0.7 was considered suitable for the reliability.^34^ In order to evaluate the stability of the CS-ICU scale, the test-retest method was conducted. The CS-ICU scale was completed by 30 patient companions in ICUs at two time with on 2-week intervals. ^35^ Then, the correlation of scores between the two tests was calculated through ICC. The ICC above 0.8 represents the appropriate stability of the questionnaire.^36^

Statistical analysis. Statistical analyses were conducted using the SPSS version 21.0. Normality data with Kolmogorov-Smirnov test was confirmed. Descriptive analysis test, factor analysis, EFA, KMO, Bartlett's Test, Cronbach's alpha, ICC and Pearson test were used for data analysis in this research. 

Ethics approval and consent to participate. The current study was approved by the ethics committee of Jahrom University of Medical Sciences in Iran with Number of Ethics IR. Jums. Rec.1397.105). the before the data collection, patient companions in ICUs signed an oral and written informed consent form. They as well as were ensured regarding the anonymity, confidentiality of the data, and voluntary participation in research.

## Results

The results of the study are presented in three phases as follows.

### The first phase

In this part, the concept of patient's companion's satisfaction in ICUs was defined based on the literature review and patient's companion's experiences using the qualitative content analysis. Patient's companion's satisfaction in ICUs is a complex and multidimensional concept which has different dimensions. The dimensions of patient's companion's satisfaction in ICUs included in four dimensions; satisfaction with nursing staff communication, satisfaction with nursing care, satisfaction with medical team personnel, and satisfaction with decision making. 

### The second phase

In this phase, the findings of the literature review and qualitative content analysis were merged in order to generate an items pool for the CS-ICU scale. The items pool consists of 85 items in four dimensions of satisfaction with nursing staff communication, satisfaction with nursing care, satisfaction with medical team personnel, and satisfaction with decision making. In the next step, the research team in three sessions reviewed the items of the CS-ICU scale for evaluate overlapping and duplicate items that finally six items were removed from the questionnaire and remained 79 items.

### The third phase

a) Face validity. In the qualitative part of the face validity, the item was not deleted and only a few items were modified based on patient's companion's comments. In the qualitative part of the face validity, eight items were deleted due to an impact score less than 1.5. Thus, 71 items remained for the CS-ICU scale.

b) Content validity. In qualitative part of content validity, five items were modified according to specialist's panel comments. In CVR assessment, the 18 items were deleted due to the CVR score of lower than 0.80. In CVI assessment, eight items were removed because of the CVI score of less than 0.79. Thus, in the end this part, 45 items remained for the CS-ICU scale.

c) The initial reliability. the internal consistency CS-ICU scale with Cronbach’s alpha was 0.93. The correlation between item number 12 “nurses provided the necessary information about the replacement of the wound dressing” with the whole CS-ICU scale was 0.01, and item number 23 “When with nurse a question is asked, they answer it” was − 0.02. Thus, the above two items deleted due to a correlation of lower than 0.3. Eventually, 43 items preserved for the CS-ICU scale.

d) Construct validity. in this section, the number of 301 patient's companions in ICUs from educational hospitals of Jahrom were completed the 43 - items CS-ICU scale in order to evaluation of EFA. The KMO value equals 0.943, which shows the appropriateness of the selected sample size in the supervision scale for the EFA. Furthermore, the Bartlett test of sphericity was significant in the level of *p* = 0.001. Hence, the data are appropriate for the factor analysis. The EFA with principal component analysis (PCA) and varimax rotation led to the extraction of three factors with Eigen value above one. Table 1 shows the Eigen value, percentage of variance for three factor and as well as factor loadings for the items that met maintenance criteria. The scree plot diagram also showed that 3 or 4 factors are sufficient to explain the concept of companion's satisfaction of patients hospitalized in ICUs from nursing services. Therefore, 21 items removed from the CS-ICU scale because of factor loading less than 0.5. Finally, 22 items and three factors remained for the CS-ICU scale. Three factors of the CS-ICU scale included the following: factor one "satisfaction with nursing staff communication" with 5 items, factor two "satisfaction with nursing care" with 12 items, and factor three "satisfaction with decision making" with 5 items. The three rotated factors explained 58% of the total variance.

The items of the CS-ICU scale were rated on a five-point Likert response scale, 1 = very low, 2 = low, 3 = moderate, 4 = high and 5 = very high. 

e) Final reliability. Cronbach's alpha of the 22-item CS-ICU scale was 0.94 that represents appropriate internal consistency. The ICC coefficient between test and retest reliability was 0.95 that indicated an optimal stability of the CS-ICU scale during the time. Also, Cronbach's alpha and ICC coefficient for three factors was calculated that are shown in Table 2.

**Table 1:** Results of a PCA of the 22 - items CS-ICU scale

subscales	Item	Factors		
		1	2	3
Satisfaction with nursing staff communication	The nurses had a good relationship with each other.	0.864		
The nurses treated with patient’s companions with respect.	0.764		
The nurses treated with patients with respect.	0.753		
The nurses established a good nonverbal communication with patients.	0.567		
The nurses answered the questions and concerns of the patients' companions properly.	0.598		
Satisfaction with nursing care	The nurses checked patients' vital signs (blood pressure, temperature, pulse and respiration) and serum status in a timely manner.		0.754	
	The nurses carefully examined the patients' problems.		0.756	
	The nurses cared for patients as a member of their family.		0.711	
	The nurses were success in face with occurrence problems in patients.		0.621	
	The nurses followed up on diagnostic procedures of patients (ultrasound, tests, photographs, CT scans, etc.).		0.599	
	The nurses provided the necessary and complete explanations about patient care at home.		0.644	
	The explanations and educations of the nurses are simple and understandable for us.		0.711	
	The nurses provided the required information for the patients' companions honestly.		0.634	
	In patients care, there was good cooperation between of the treatment team members.		0.588	
	The nurses paid attention to the privacy and culture of the patients during care.		0.521	
	The nurses paid attention to patients' emotional needs (such as the meeting patients' needs) and responded appropriately.		0.577	
	The nurses responded to patients' religious-spiritual needs appropriately (such as the call to prayer).		0.579	
**Satisfaction with decision making**	The nurses were involved the patient's companions in the care process.			0.722
	The nurses were involved the patient's companions in decision-making processes for patients in times of need.			0.635
	The nurses informed the patient's companions of the decisions made for the patient if needed.			0.612
	The nurses agreed with the patient's companions about care and treatment methods of patients if needed.			0.533
	The nurses supported from patients' companions suggestions about patients care methods.			0.512
Eigen value		5.301	3.289	1.987
Percentage of variance		23.345	19.450	15.205

**Table 2:** The Cronbach's alpha and ICC values for CS-ICU scale and its factors

Factors	Subscales	Number of items	Internal consistency	Stability
1	Satisfaction with nursing staff communication	5	α = 0.92	ICC = 0.96
2	Satisfaction with nursing care	12	α = 0.92	ICC = 0.91
3	Satisfaction with decision making	5	α = 0.89	ICC = 0.94
Total	CS-ICU scale	22	α = 0.94	ICC = 0.95

## Discussion

The present study dealt with the development and validation of a scale for patient's companion's satisfaction in ICUs. Patient's family and companion's satisfaction is one of the important criteria in assessing quality of care in ICUs.^20^^,^^37^ Measuring companion's satisfaction of the patients hospitalized in ICUs is significant since most of the ICUs patients can't make decision about their care; as well as assessing patient's companion's satisfaction can help the improvement procedure of services, cares and provided treatments.^38^ The result showed that the CS-ICU scale was a reliable and valid questionnaire for the evaluation of patient's companion's satisfaction in ICUs**.**

There are several questionnaires to assess the patient's companion's satisfaction in ICUs. Only four instruments could be classified as being of “well-established quality”: the CCFNI, the SCCMFNA, the CCFSS, and the FS-ICU. Nevertheless, these high-quality questionnaires consisted of 35 different versions, each with large disparities in psychometric properties.^1^^,^^3^ However, these questionnaires have limitations. The limitations of the instruments include insufficient data regarding (1) construct and content validity (2) inter-rater reliability and (3), test-retest reliability (1). Due to construct validity is the extent to which a questionnaire actually measures what it claims to measure, and content validity refers to whether the tool includes the proper information, they both are of great importance, especially in a subjective outcome like satisfaction. Differences may arise because inherent semantic differences and socio-cultural differences. For example, the degree of companions and family participation in the decision-making process differs across the world.^39^ Therefore, in order to accurately measure of satisfaction, a specific questionnaire be tailored to the socio-cultural and context - based conditions of the same community is required.

The developed and validated questionnaire of CS-ICU in this study had three domain including satisfaction with nursing staff communication, satisfaction with nursing care, and satisfaction with decision making with 22 items. These three domains are central to overall companions and family satisfaction with ICU care. First, satisfaction with nursing care provides information on how families and patients companions experience general aspects of care. Second, patient's companion's satisfaction with decision making is an important element due to the family and companions is a substitute decision maker for their especially ill family member in a complex healthcare environment such as ICUs. Patient's companion's satisfaction is also related to the family being provided with clear data due to this enables them to actively participate in the decision-making process.^1^^,^^3^^,^^40^ The FS-ICU scale with 34 - items were developed by Heyland and Tranmer^41^ included two domains of satisfaction with care and satisfaction with decision making. Some items of the satisfaction with care and satisfaction with decision making domains in the FS-ICU scale are comparable with items of the CS-ICU scale with 22 - items in the present study. The items in the FS-ICU scale were derived from the existing literature on patient satisfaction and quality of care near the end of life. However, the items in the CS-ICU scale were derived from the existing literature on patient satisfaction and interview with companions of patients hospitalized in ICUs. 

The CCFSS developed by Wasser *et al.*^42^ is another questionnaire to measure family satisfaction with intensive care that included five domains of assurance, information, proximity, support, and comfort. The result of studies about CCFSS as well as shows first in five studies^42^^-^^46^ reported adequate internal consistency, whereas four other studies^47^^-^^50^ found it to be poor. Second, CCFSS had mediocre responsiveness and data on other psychometric data are lacking. Third, a questionnaire designed in a particular country only reflects the language and culture of the same community, in which case due to the content inconsistency it will cause many problems when used in another community.^51^ The quality of a questionnaire is as well as highly dependent on the circumstances under which it is used. In addition to, it depends on what population it is used on. For example, differences in language, culture, and patient companion's population have a high effect on the appropriateness of a questionnaire.^1^^,^^51^ Also, the SCCMFNA 14 - items scale developed by Johnson *et al.*^52^ and CCFNI 45 - items scale developed by Molter ^53^ both measures need of family members which differs from the purpose of satisfaction measurement in the present study. In this regard, Heyland *et al*.^54^ express that although satisfaction reflects the amount of fulfillment of needs and expectations, but meeting needs does not guarantee satisfaction.

Finally, in the evaluation of family and companions’ satisfaction with intensive care, the use of valid and reliable questionnaires is essential to gain appropriate and high-quality data. Nevertheless, this is the first study in Iran that critically examined the psychometric properties of companion’s satisfaction questionnaires of patients hospitalized in ICUs. This data is necessary as an outcome quality indicator and to better target improvement initiatives in the ICU. One of the strengths of present study is that the CS-ICU scale was developed the both inductive and deductive approach and as well as have been used psychometrics properties consist of face, content, and construct validity, internal consistency and test - retest reliability. Also, the CS-ICU scale is a short-form (22 items) questionnaire that can be responded by patient's companions in about 10 minutes, which is indicating the feasibility of using this questionnaire. The greatest strength of the present study was the development of a context-bound questionnaire to assess companion's satisfaction of patients hospitalized in ICUs. Besides the strengths of the described above, this study also holds limitations. First, we do not know the opinions and comments of non-participating families and companions. Another limitation of this study is that the questionnaire suffering of self-report scales.

Conclusion. In the present study, the three-dimension CS-ICU was developed as a short self-report scale for measurement of companion's satisfaction of Iranian patients hospitalized in ICUs. The CS-ICU scale is a valid, reliable and context-based questionnaire which can be used in different levels in the healthcare centers such as education, research, care management, satisfaction assessment, and improving nursing services.

Availability of data and material. Data is not and will not be made available elsewhere. Further data set could be obtained on request if required through corresponding author with email: ali.dehghani2000@ gmail.com.
